# Evaluating longitudinal relationships between parental monitoring and substance use in a multi-year, intensive longitudinal study of 670 adolescent twins

**DOI:** 10.3389/fpsyt.2023.1149079

**Published:** 2023-05-12

**Authors:** Jordan D. Alexander, Samantha M. Freis, Stephanie M. Zellers, Robin Corley, Amy Ledbetter, Rachel K. Schneider, Chanda Phelan, Hariharan Subramonyam, Maia Frieser, Gianna Rea-Sandin, Michelle E. Stocker, Helen Vernier, Ming Jiang, Yan Luo, Qi Zhao, Sally Ann Rhea, John Hewitt, Monica Luciana, Matt McGue, Sylia Wilson, Paul Resnick, Naomi P. Friedman, Scott I. Vrieze

**Affiliations:** ^1^Psychology Department, University of Minnesota, Minneapolis, MN, United States; ^2^Institute for Behavioral Genetics, University of Colorado Boulder, Boulder, CO, United States; ^3^Institute for Molecular Medicine Finland, University of Helsinki, Helsinki, Finland; ^4^School of Information, University of Michigan, Ann Arbor, MI, United States; ^5^Graduate School of Education, Stanford University, Palo Alto, CA, United States; ^6^Department of Computer Science, University of Minnesota, Minneapolis, MN, United States; ^7^Institute of Child Development, University of Minnesota, Minneapolis, MN, United States

**Keywords:** cannabis, alcohol, adolescence, GPS, parental monitoring, behavioral genetics, intensive longitudinal assessment

## Abstract

**Introduction:**

Parental monitoring is a key intervention target for adolescent substance use, however this practice is largely supported by causally uninformative cross-sectional or sparse-longitudinal observational research designs.

**Methods:**

We therefore evaluated relationships between adolescent substance use (assessed weekly) and parental monitoring (assessed every two months) in 670 adolescent twins for two years. This allowed us to assess how individual-level parental monitoring and substance use trajectories were related and, via the twin design, to quantify genetic and environmental contributions to these relationships. Furthermore, we attempted to devise additional measures of parental monitoring by collecting quasi-continuous GPS locations and calculating a) time spent at home between midnight and 5am and b) time spent at school between 8am-3pm.

**Results:**

ACE-decomposed latent growth models found alcohol and cannabis use increased with age while parental monitoring, time at home, and time at school decreased. Baseline alcohol and cannabis use were correlated (*r* = .65) and associated with baseline parental monitoring (*r* = −.24 to −.29) but not with baseline GPS measures (*r* = −.06 to −.16). Longitudinally, changes in substance use and parental monitoring were not significantly correlated. Geospatial measures were largely unrelated to parental monitoring, though changes in cannabis use and time at home were highly correlated (r = −.53 to −.90), with genetic correlations suggesting their relationship was substantially genetically mediated. Due to power constraints, ACE estimates and biometric correlations were imprecisely estimated. Most of the substance use and parental monitoring phenotypes were substantially heritable, but genetic correlations between them were not significantly different from 0.

**Discussion:**

Overall, we found developmental changes in each phenotype, baseline correlations between substance use and parental monitoring, co-occurring changes and mutual genetic influences for time at home and cannabis use, and substantial genetic influences on many substance use and parental monitoring phenotypes. However, our geospatial variables were mostly unrelated to parental monitoring, suggesting they poorly measured this construct. Furthermore, though we did not detect evidence of genetic confounding, changes in parental monitoring and substance use were not significantly correlated, suggesting that, at least in community samples of mid-to-late adolescents, the two may not be causally related.

## 1. Introduction

Adolescence is a time of rapid psychological, developmental, and environmental change that is frequently characterized by increases in autonomy, exploration, and risk-taking behaviors (1). Correspondingly, many youths begin to experiment with drugs and alcohol during this period (2). A majority of late adolescents report that alcohol and cannabis are easily obtainable as their use is often culturally sanctioned and, in many US states, they can be legally obtained by slightly-older peers (3, 4). Along with these clear environmental influences on the availability of addictive substances, individual differences in adolescent substance use are also influenced by genetic factors (5, 6).

While developmentally normative in United States adolescents (3), substance use places youth at increased risk for a multitude of adverse consequences. Adverse outcomes include long term health consequences like increased risk for cardiovascular diseases, cancers, and future substance use disorders, as well as more immediate consequences like physical injuries, impaired judgment, risky sexual behaviors, legal consequences, and even accidental death (7, 8).

To protect against these risks, numerous behavioral interventions have been developed to prevent or reduce adolescent substance use. Parent management training programs, such as Parent Management Training—Oregon Model (PMTO), are among the most popular of these interventions (9, 10). These interventions are premised on the coercion model of delinquency, in which parents who initially adopt harsh or coercive parenting practices evoke problem behaviors from their children and subsequently respond to these behavior problems by disengaging from supervising their children (9). The combination of these evoked behavior problems and subsequent decreases in parental monitoring are then hypothesized to promote further delinquent behaviors, like engaging in substance use. PMTO programs therefore attempt to pre-empt this trajectory by intervening to foster more positive parent–child interactions and to increase parental monitoring of children’s activities. Such programs have proven to be reasonably effective in reducing adolescent substance use, with recent meta-analytic estimates suggesting a small to moderate effect of parent management training on adolescent substance use (10). Furthermore, several studies report robust negative associations between parental monitoring and adolescent substance use, with several showing longitudinal associations wherein parental monitoring in childhood and adolescence predicted substance use in emerging adulthood (11–13).

However, this conceptualization of parental monitoring has been challenged by subsequent research. While early conceptualizations of the relationship between parental knowledge and substance use posited that the relationship represented the effects of parental surveillance or control of adolescent activities on reducing delinquent behavior (14), a series of studies by Kerr and Stattin on the relationship between parental monitoring and delinquency in Swedish youth demonstrated that adolescents’ willingness to disclose information about their activities to their parents was more predictive of both parental knowledge and subsequent delinquency than were active parental surveillance efforts (15–18). Furthermore, Eaton et al. found that the relationship between both parental knowledge and adolescent disclosure was largely accounted for by adolescent personality, suggesting that pre-existing differences in adolescent personality, such as one’s willingness to disclose information to their parents, explain previously observed relationships between parental monitoring and delinquent behaviors.

Additionally, studies supporting the relationship between parental monitoring and delinquent behaviors like substance use universally rely on correlational research designs conducted in cross sectional or sparse longitudinal data. Such designs are poorly suited to the causal claims inferred from them, as a correlation may arise from a causal relationship between two variables, but it may also be due to any number of other factors influencing both variables (19, 20). An additional complication is that commonly assessed, putatively environmental risk factors for behavioral traits are often heritable themselves (21). This highlights the importance of gene–environment correlation, where heritable attributes of individuals affect the environmental experiences they have. Genetically informed research designs have often found that relationships between alleged environmental risk factors and behavioral outcomes are largely explained by pre-existing impacts of genetics (22). In the case of parental monitoring, Eaton et al.’s finding that correlations between parental monitoring and substance use appear to be largely attributable to adolescent personality traits, is suggestive that their relationship could reflect gene–environment correlation, wherein genes influencing substance use also influence the degree of parental monitoring one experiences. For example, adolescent control, a highly heritable personality trait, may have led to both increased parental disclosure and reduced substance use, thereby confounding the correlation between parental monitoring and substance use. Such findings highlight the role of gene–environment correlations in the relationship between behavioral traits and hypothesized environmental risk factors, necessitating the use of genetically informative samples to measure and control for these confounds.

Furthermore, parental monitoring, like other environmental risk factors for behavioral outcomes, is traditionally assessed via self-report. Such questionnaires can be effective, but limitations exist over and above issues such as recall bias (23). Ambulatory assessment using wireless devices—typically smartphones—represent a newer and relatively untested approach to evaluating behaviors or environmental exposures quasi-continuously over time (24). Questionnaires can be administered on the device at any time and any interval. Additional “passive” data can be collected on a participant’s location, movement and, depending on the sensors available, biological attributes such as cardiovascular or respiratory function (25). These technological developments may facilitate novel measurement paradigms to supplement or even replace self-report inventories (26), but, while preliminary research has been promising (25, 27), the feasibility of passively collecting valid and useful psychological data is less clear.

We thus attempted to address some of these challenges using data from the CoTwins sample, an ongoing intensive longitudinal twin study of adolescent substance use conducted at the University of Colorado and the University of Minnesota. In the CoTwins study, 670 twins and their parents were assessed in-person at an intake assessment, at which time an app was installed on each twin’s smartphone. They were then assessed remotely via the smartphone app for 2 years. We used the app to administer regular questionnaires including measures of alcohol use, cannabis use, and parental monitoring. The app also passively monitored geographical location, which we used to infer whether an individual was at home or at school during days/times when one would expect an adolescent to be at home or at school. We hypothesized that these geospatial measures would measure discordance between a twin’s actual and expected location during these hours and would thus offer additional information on the parental monitoring construct. Parents of adolescents who, for example, were frequently out of the home late at night or were away from school during school hours were hypothesized to have a lower degree of parental knowledge than parents of adolescents who were generally at home or at school during these times. Correspondingly, under the coercive model of parenting leveraged by some popular substance use interventions (9, 28), such adolescents would be expected to exhibit greater rates of delinquent behaviors like substance use.

These quasi-continuous locations were analyzed along with the questionnaire data to characterize adolescent change in these domains from ages 14 to 18. These data allowed us to (1) replicate the expected correlation between parental monitoring and substance use, (2) evaluate whether change in adolescent substance use is associated with change in parental monitoring during adolescence, (3) determine whether simple GPS-derived measures of location could serve as proxies for parental monitoring or environmental risk for substance use, and (4) using the genetically informative twin design, determine whether relationships between substance use and parental monitoring trajectories were confounded by mutual genetic influences.

In doing this, we offer a further test of the hypothesis that parental monitoring is causally related to adolescent behavioral problems like substance use. If changes in parental monitoring correspond with changes in substance use above and beyond what can be explained by genetic confounding effects, this study will bolster support for a possible causal relationship between them. However, if changes in parental monitoring correspond poorly with changes in substance use or if the relationship appears largely driven by gene–environment correlation, then evidence for a causal relationship between the two would be significantly challenged, as would the role of parental monitoring as a target in substance use interventions.

## 2. Materials and methods

### 2.1. Participants

Participants were adolescent twins who were recruited to participate in the CoTwins study in 2015 and 2016. These participants were recruited using the Colorado Twin Registry, a population-based registry of twins born in Colorado. Families were eligible if they had twin children between the ages of 14 and 17, who had Android or iOS smartphones, and who resided in Colorado or nearby states. The study was approved by the University of Colorado Boulder and University of Minnesota Institutional Review Boards.

Informed consent and assent were obtained from both parents and children. The sample consisted of 109 monozygotic (MZ) twin pairs (67 female and 42 male pairs) and 221 dizygotic (DZ) twin pairs (71 female, 63 male, and 87 opposite-sex pairs). Their ages at recruitment were between 14 and 17 (mean: 16.1; SD: 1.1); their race and ethnicities, as described by their parents, were 71% non-Hispanic white, 14% Hispanic/Latinx, 10% multi-racial, and 3% of other ethnicities. At the baseline visit, 73% of participants reported that their parents were still married while 27% reported a parental divorce. At the time of their first remote survey, 73% of twins reported living with both their mother and father, 5% reported an additional adult in the house, 5% reported living with at least one stepfather or stepmother, and 16% reported living with only one guardian: either their mother, father, or another adult.

### 2.2. Procedure

Following recruitment, we conducted an intake visit where the twins’ zygosity was determined and twins and their parents completed baseline assessments including measures of parental monitoring and substance use. During the visit, the CoTwins software application (the “app”) was installed on the twins’ phones, for iOS and Android operating systems. The app was then used to regularly administer remote assessments to the twins, but not to their parents, for the duration of their participation in the study. Remote questionnaires were adapted from the in-person measures to help ensure comparability while minimizing participant burden. Data analyzed in this manuscript were collected by the app between May of 2015 and November of 2018. Initially, twins participated for 1 year of remote assessment via the app. Seventy-nine percent of twins agreed to a second year.

In addition to administering surveys, the app also collected global positioning system (GPS) latitude/longitude and time stamp data, with collection density/accuracy calibrated to guard against substantial battery drain. Android and iOS location modules are “black boxes,” which perform sensor fusion and produce location estimates in unknown and proprietary ways. On iOS, we used the significant change location API and locations were recorded only when the user moved a “significant” distance and no more frequently than every 5 min. On Android, the user’s location was recorded every 5 min. On an approximately weekly basis, study staff monitored questionnaire completion rates and passive data collection and contacted twins to offer technical support when necessary. Before data cleaning, the median accuracy, as reported by the location API, was 65 m. After removing locations with an accuracy worse than 500 m, it was 17.1 m.

### 2.3. Measures

#### 2.3.1. Substance use

Substance use questions were derived from the Substance Abuse and Addiction collection of the PhenX Toolkit, a set of reliable and well-validated substance-abuse related measures that have been made publicly available to improve the harmonization of substance use measurement across research studies (29, 30) though, due to low base rates of other substance use, only alcohol and cannabis use were included in subsequent analyses (30, 31). Past week alcohol use frequency was assessed by the question “In the last 7 days, since last [assessment date], on how many days did you drink any alcohol?” Alcohol quantity per use occasion was assessed via the question “On those days that you drank alcohol, how many drinks did you usually have each day? (One “drink” is equal to 1 can or bottle of beer, a glass of wine, or a shot of hard liquor.)”

Similarly, marijuana use frequency was measured by the item “In the last 7 days, since last [assessment date], on how many days did you use any marijuana or hashish, including smoking marijuana, edibles, vaping, dabbing, or however else you may have used marijuana?” Marijuana quantity, measured as the number of times per day a participant used marijuana on a typical day in which they used marijuana, was assessed via the question “On each day that you used marijuana (whether smoked, eaten, vaped, dabbed, or however it was used), how many times per day did you use enough to feel the effects?”

Substance use was assessed every 3–7 days (uniformly distributed with mean = 5) until May, 8th, 2017, roughly 2 years after the start of data collection, when the frequency was changed to 5–9 days (uniform with mean = 7) to further reduce participant burden. On average, participants completed 49.7% of substance use assessments which they were administered, completing an average of 62.1 assessments over the duration of the study. Weekly substance use quantity-frequency was calculated for alcohol (as drinks per week) and cannabis (as cannabis use occasions per week). These were then log-transformed (after adding 1 to keep zeros) to reduce the influence of outliers on model results and so that parameter estimates would represent relative changes rather than absolute changes in the outcome variable (32). Descriptive statistics, including ICCs and Cronbach’s α’s, for the substance use variables, parental monitoring and geospatial measures, are presented in Table 1.

**Table 1:** Descriptive statistics of substance use, parental monitoring, and geospatial variables.

	Total responses	Mean responses per participant	Grand mean	Grand SD	Mean (aggregated within subjects)	SD (aggregated within subjects)	ICC	Cronbach’s α
Alcoholic drinks per week	40,923	62.1	0.45	2.43	0.51	1.54	0.37 [0.35, 0.40]	0.97 [0.96: 0.98]
Marijuana uses per week	40,919	62.1	0.25	1.68	0.35	1.76	0.61 [0.58, 0.63]	0.98 [0.98: 0.99]
Parental monitoring	5,925	9.0	16.40	3.21	16.43	2.58	0.67 [0.65, 0.70]	0.92 [0.83: 0.87]
Time spent at home	14,872	40.2	0.63	0.30	0.67	0.19	0.33 [0.30, 0.37]	0.97 [0.96: 0.98]
Time spent at school	9,637	25.1	0.50	0.28	0.51	0.19	0.36 [0.33, 0.40]	0.94 [0.94: 0.96]

Total responses are the number of total observations recorded for a measure. Mean responses per participant are computed as the total number of responses divided by the number of contributing participants. Grand means and standard deviations (SDs) are the mean and standard deviation for each measure disaggregated across participants. The mean and SD aggregated within subjects are the mean and standard deviation for the measure after aggregating observations within subjects (i.e., computing within subject means). To ease interpretation, alcoholic drinks per week and marijuana uses per week are not presented on a log scale here, though they are log transformed within the models. Parental monitoring is measured as the sum of the items on the adolescent-reported parental monitoring questionnaire for the parent with the highest parental monitoring score at that timepoint. Time spent at home is defined as the proportion of time spent within 100 meters of one’s home address between 12AM and 5AM on a given day, while time spent at school is defined as the proportion of time spent within 200m of a Colorado High School between 8AM and 3PM on a given day. Intraclass coefficients (ICC) are the single random raters ICC and measure the average correlation between pairs of observations on each measure. Cronbach’s α are measured longitudinally, with each response representing an item in a “scale” comprising all of an individual’s responses over the duration of the study.

#### 2.3.2. Parental monitoring

As no measure of parental monitoring was available from the PhenX toolkit at the time of analysis, parental monitoring questions were obtained from a parental monitoring questionnaire developed by the Minnesota Center for Twin and Family Research (33, 34). Prior research using this questionnaire has established that both the parent and adolescent reported parental monitoring measures and their subscales are reliable and associated with related constructs like adolescent personality and delinquent behaviors (34). At baseline, parental monitoring was assessed along with parental solicitation (the extent to which parents ask about their children’s activities) and parental disclosure (the degree to which adolescents share information about their activities) using a 15-item parental monitoring questionnaire, with twins completing the adolescent-report version and their parents completing an analogous parent-report version. To avoid artificial depression due to, for example, single parent families, the maximum values for each question (most knowledge of the child’s activities) were chosen across all parental figures for that twin. Then, maximum values were summed across questions to produce a sum score. Questions on this form included items assessing the degree to which parents were aware of, solicited information on, or were told where and with whom adolescents spent their time.

The baseline measures of parental monitoring differed from the measure which were administered remotely to the adolescents after the baseline visit. Hence, these baseline measures of parental monitoring were not included in subsequent analyses of relationships between parental monitoring and substance use, except as a means of testing the validity of our remote parental monitoring measure. After the baseline visit, adolescent-reported parental monitoring was administered remotely using only the parental knowledge subscale from the in-person parental monitoring questionnaire. The parental knowledge items were chosen to represent the parental monitoring construct as parental knowledge has been found to be more predictive of adolescent substance use than either parental solicitation or adolescent disclosure, likely because it includes information parents have obtained through both processes (18, 34). These consisted of five items per parent/guardian. This questionnaire was administered to the twins randomly every 50–70 days (uniform with mean = 60). On average, participants completed 92.4% of remote parental monitoring assessments, completing an average of 9.1 assessments over the duration of the study. The five questions used to assess parental monitoring were (1) “My [parent] knows who I spend time with,” (2) “My [parent] knows how I spend my money,” (3) “My [parent] knows where I am most afternoons after school,” (4) “My [parent] knows where I go at night,” and (5) “My [parent] knows what I do with my free time.” These items were rated on a five-point scale from “never” to “always.” Twins were asked to respond to these five questions for each of the adult parental figures they lived with. As on the in-person assessment, parental monitoring scores were computed, by selecting the highest value on each item across all parental figures and summing these items to produce a sum score. These remote parental monitoring questions are included as a supplement to the manuscript.

At intake, adolescent-reported parental monitoring was highly correlated with adolescent-reported parental disclosure (*r =* 0.72) and moderately correlated with adolescent-reported parental solicitation (*r* = 0.46). Adolescent-reported monitoring was more modestly correlated with parent-reported monitoring (*r =* 0.35) and disclosure (*r =* 0.22) but was not correlated with parent-reported solicitation (*r =* 0.00). At intake, adolescent reported parental monitoring measures were reasonably reliable as individual subscales (Cronbach’s alphas = 0.77–0.79) and when aggregated together (Cronbach’s alpha = 0.86) suggesting these scales were measuring related constructs. Similarly, baseline parent-reported parental monitoring measures were also reliable both as individual scales (Cronbach’s alphas = 0.83–0.86) and in aggregate (Cronbach’s alpha = 0.86). Adolescent-reported parental knowledge and parental disclosure at baseline were both significantly correlated with alcohol (*r*s = −0.13 to −0.09) and marijuana use (*r*s = −0.22 to −0.20) at the first remote follow up while adolescent-reported parental solicitation was not significantly correlated with either substance use measure (*r*s = −0.04 to 0.00). Similarly, baseline parent-reported parental knowledge and adolescent disclosure were significantly correlated with first-follow-up alcohol (*r*s = −0.18 to −0.13) and marijuana use (*r*s = −0.19 to −0.29) while parent-reported solicitation efforts were only significantly associated with alcohol use (*r =* 0.09).

The rank correlation between the intake in-person parental monitoring assessment and the first remote follow-up assessment, approximately 1 month later, was 0.57 (95% CI = 0.48–0.61) for the adolescent-report form and 0.35 (95% CI = 0.28–0.41) for the parent-report form. Cronbach’s alpha for the first remote follow-up parental monitoring score was 0.75 (95% CI = 0.72–0.78). Remote parental monitoring assessments had an intraclass correlation coefficient of 0.68, indicating moderate correspondence between repeated measures over time.

#### 2.3.3. Geospatial measures

To facilitate analyses, prior to computing geospatial measures, each twin’s GPS locations were first standardized into a series of consecutive, 30-min time windows, starting at their first recorded point and ending at their last recorded point. For each twin, the GPS location within each window closest to the center of that window was chosen to represent the window and produce a standardized point. Next, we accounted for the fact that the iOS application only records a point when the user has moved more than ~500 m, by filling forward missing standardized iOS points for up to 12 h. The 12 h period was chosen as a commonly expected duration with no movement, such as an over-night stay at home. On average, after data cleaning and fill-forward procedures, participant location was reported for at least part of the day on 76.2% of days, with at least one point recorded for 50% of possible 30-min windows for the duration of the study.

For estimates of time at home, a filled and standardized point was considered “at home” if it was within 100 m of any of the geocoded home addresses on file for that family. Then, the fraction of points at home between midnight and 5 AM was calculated each week, for each twin, and this fraction was used as the “time at home” variable. If a manual inspection showed that a twin was consistently never at home, we inferred that we had an incorrect home address and removed them from the at home data.

For time at school, a list of public and private schools in the state of Colorado was downloaded from the ElSi Table Generator maintained by the National Center for Education Statistics.^1^ The latest relevant data release was used, from the 2015 to 2016 school year. High schools were selected, and the physical address of each school was geocoded. A filled and standardized point was considered “at school” if it was within 200 m of any of the schools in the list. Then, those points were subset to include only school hours (8 AM–3 PM) and school days, as determined by Colorado public school calendars and manual review of the location data. Time at school was then defined as the fraction of remaining points at school each week, for each twin. If a manual inspection showed that a twin was consistently never at school, we concluded that their school was not included in the ElSi database or that they were home schooled and set their time at school to missing.

#### 2.3.4. Zygosity

Twin pairs were rated as either monozygotic (MZ), same sex dizygotic (DZ), or opposite sex dizygotic (OS). OS twins were automatically rated as dizygotic as there are no opposite sex MZ twins. For same sex twins, zygosity was determined by two expert coders, who independently assessed twin similarity on six physical traits on a five point similarity scale. Discrepancies between raters were resolved via discussion before arriving at a consensus zygosity determination.

### 2.4. Analyses

To characterize average longitudinal phenotypic trajectories (i.e., mean change during adolescence) for time at home, time at school, parental monitoring, drinks per week, and cannabis uses per week, non-linear mean functions were estimated using generalized additive mixed models (GAMMs) fit by the R package gamm4 (35). The phenotype of interest was predicted by smooth functions of age, which were fit by penalized regression with sex as a covariate. The basis dimension for each phenotype was chosen using the residual randomization test implemented in the R package mgcv (36) with the random effects for each smooth term nested by twins within twin pairs.

To understand individual differences (i.e., variance) in the developmental trajectories of parental monitoring and substance use and to estimate the genetic and environmental contributions to these differences, we utilized a multivariate growth modeling approach. We expect that, after age 18, when many adolescents complete high school and leave the home, the meaning of the parental monitoring and geospatial phenotypes and their relationships to substance use will change. Therefore, to avoid these likely confounds, all assessments after age 18 were removed before fitting these multivariate latent growth models. The models were fit using the R package OpenMx (37, 38). Missing observations were addressed via full information maximum likelihood estimation.

To represent individualized developmental trajectories in each phenotype as a function of participant age, we considered structural equation models predicting each outcome as a function of a random intercept, random effect of age (slope), and random effect of age squared (acceleration). The inclusion of linear and quadratic age random effects was based on initial models run in the R package lme4 (39), where models with random intercepts and age slopes at both the individual and family level were compared to models with random intercepts, age slopes, and age quadratic terms. Cubic models were considered as well but were ultimately not selected due to those models failing to converge in lme4. Based on AIC and BIC criteria, models which included random age slopes and quadratic terms offered superior fit for all five phenotypes considered.

To render intercepts and slopes more interpretable, age and age^2^ were scaled so that a value of 0 corresponded to age 14. To measure correlations between growth parameters (e.g., the correlation between the random alcohol slopes and random parental monitoring slopes) each model included two of the five measures under study (alcohol use, cannabis use, parental monitoring, time spent at home, and time spent at school). When jointly modeling substance use variables with parental monitoring, 10 total models were implemented, representing all possible combinations of these five outcomes.

To assess the additive genetic (A), shared environmental (C), and nonshared environmental (E) contributions to these growth parameters and the degree to which genetic and environmental influences are shared between growth parameters, we decomposed the random effects and residuals into ACE components. ACE models leverage the difference in genetic relatedness between MZ twins, who share 100% of segregating genes, and DZ twins, who share on average 50% of segregating genes, to estimate the genetic and environmental contributions to a phenotype (or the covariance between two phenotypes). Additive genetic effects (A) represent the influence of genetic variation on phenotypic variation and are identified when MZ twins are more alike than DZ twins. Shared environmental effects (C) represent elements of the environment that increase the similarity of twins in the same family and are identified when MZ twins are less than twice as phenotypically similar to one another as DZ twins (because MZ twins are twice as genetically similar as DZ twins, MZ twins are expected to be twice as similar as DZ twins in the absence of shared environmental influences). Non-shared environmental effects (E) represent elements of the environment that lead to differences between members of the same family and are identified when MZ twins are not perfectly correlated with one another.

**Figure 1:**
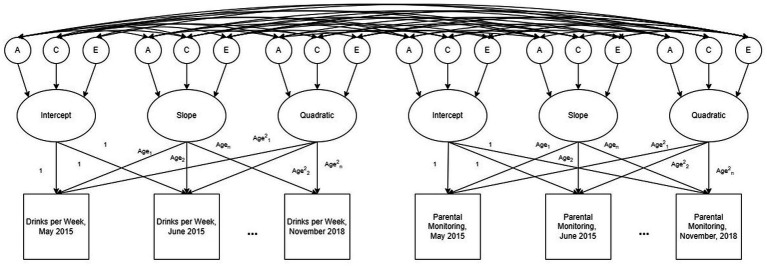
An example path diagram representing the ACE decomposed multivariate latent growth model for drinks per week and parental monitoring. The model estimates random effects, including intercepts, age slopes, and age quadratic terms, for each participant as well as the additive genetic (A), shared environmental (C), and non-shared environmental (E) contributions to these random effects. Genetic correlations (*r*_g_), shared environmental correlations (*r*_c_), and nonshared environmental correlations (*r*_e_) between random effects terms are represented by the paths between the A, C, and E terms. Additional bivariate models were run comparing drinks per week, cannabis use per week, perceived parental monitoring, time spent at school, and time spent at home.

Confidence intervals for the variances and covariances of the random intercepts, slopes, and quadratic terms and their ACE variance components were obtained using likelihood-based confidence intervals implemented in OpenMx. To provide readers with additional clarity on the structure of these models, an example path diagram of the model comparing drinks per week and parental monitoring is provided in Figure 1.

Together, these ten growth models were used to estimate a 15 × 15 ACE decomposed variance–covariance matrix of the random intercepts, slopes, and quadratic terms estimated in the models. Cross-phenotypic correlations between the random intercepts were used to measure whether variables were correlated at age 14 while those between the random slopes and quadratic terms measured whether developmental changes in a phenotype (e.g., parental monitoring) were associated with corresponding changes in another phenotype (e.g., substance use). The ACE decompositions of the variances were used to measure the genetic and environmental contributions to the initial levels and developmental changes in each phenotype, such as whether the development of parental monitoring or substance use is heritable. Lastly, the ACE decompositions of the covariance terms was used to estimate the degree of genetic and environmental correlation between the growth parameters of each phenotype, such as whether parental monitoring and substance use share genetic or environmental influences.

To assess whether the sample was sufficiently well powered to identify random effects correlations, ACE, parameters, and biometric correlations, a number of post-hoc power analyses were conducted. Power analyses for correlations between growth parameters were conducted via simulation. Using the “mvrnorm” function from the MASS package in R (40), data were simulated for each of the 10 combinations of phenotypes in samples of 670 participants measured at 24 timepoints, representing a full two-year participation period in the study. Data were simulated as arising from a bivariate growth model with random intercept, slope, and quadratic effects that were allowed to correlate across phenotypes. Random effects terms were generated with means of 0 and variances taken from the results of the original ACE-decomposed latent growth-curve models (presented in Supplementary Table S1). To account for missing data, 50% of observations were set as missing. Bivariate growth models analogous to those from the primary analysis, but without ACE components, were then fit to each simulated dataset. Simulations were repeated 100 times while varying correlations between random intercept, slope, and quadratic parameters to determine the minimum value of each random effects correlation that could be detected 80% of the time at an alpha level of 0.05. Eighty percent power to detect phenotypic correlations was achieved for 13/30 parameters at *r =* 0.15, for 20/30 parameters at *r = 0*.*25*, and for 25/30 parameters at *r = 0.35*. Lastly, power analyses for standardized multivariate ACE components were conducted via simulation using the “powerFun” functions described in Verhulst (41). Eighty percent power to detect genetic variance components was achieved at A = 0.40 when C = 0 and at A = 0.34 when C = 0.2. Similarly, 80% power was achieved to detect C variance components of C = 0.22 or greater with moderate genetic effects, A = 0.50 and at C = 0.25 when genetic effects were assumed to be small (e.g., A = 0.30). Assuming modest shared environmental influences of C = 0.20, models achieved 80% power to detect genetic correlations, the degree to which genetic influences are shared between two traits, when *r_g_* = 0.26 between highly heritable traits (A = 0.70) and when *r_g_* = 0.72 for moderately heritable traits (A = 0.5). Genetic correlations between more modestly heritable traits (A = 0.30) could not be reliably detected, achieving only 18% power even when *r_g_* = 1.00.

## 3. Results

### 3.1. Mean phenotypic trajectories

During the remote assessment period, the rate of survey completion was consistent during the first year, with some decline during the second (Supplementary Figure S1A). The most frequently used substances in these assessments were alcohol (use reported in 6.9% of measurement occasions) and cannabis (use reported in 5.0% of measurement occasions).

**Figure 2:**
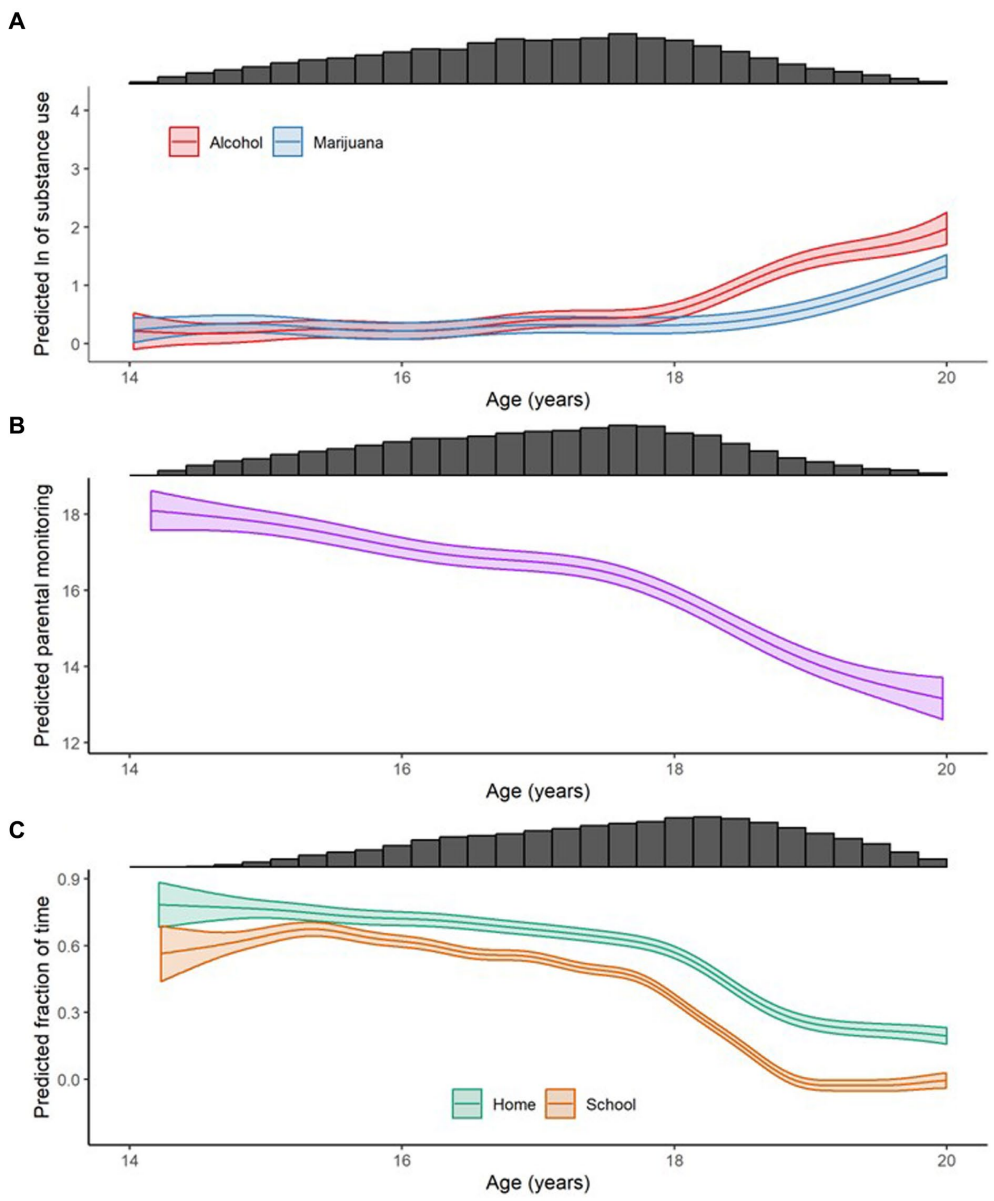
Smoothed means (on log scale) conditional on age, as calculated with generalized additive mixed models, of **(A)** natural log-transformed ("ln") drinks per week (Alcohol), cannabis uses per week (Cannabis), and e-cigarette uses per week (E-Cigarettes); **(B)** parental monitoring; and **(C)** the fraction of time spent at the family home at night (Home) and the fraction of time spent at school during the school day (School). Uncertainty in the estimate is shown as 95% confidence intervals and the marginal histograms show the relative number of data points available for a given phenotype in a given age range.

Average developmental trajectories in substance use, parental monitoring, and the geospatial variables, measured via GAMMs, are presented in Figure 2. Both alcohol and cannabis use (Figure 2A) increased with age, with rapid acceleration after age 18, even on the log scale. The mean trajectory of parental monitoring (Figure 2B) demonstrated the expected decrease of parental monitoring with age; the decrease accelerated after age 18. Time at home and school estimated from GPS recordings (Figure 2C), both decreased with age, with rapid decreases between age 18 and 19 and relatively little change after age 19. Overall, these mean trajectory plots show average developmental increases in substance use and decreases in parental monitoring and geospatial measures as participants aged, with clear inflection points in all phenotypes around age 18.

### 3.2. Parental monitoring and substance use trajectories

The phenotypic correlations between participants’ growth parameters in the latent growth models (intercepts, age slopes, and age quadratic terms) are reported in Table 2 while their covariances are presented as a supplement in Supplementary Table S1. Moderate to large positive associations were identified between substance use growth parameters: the random intercepts (*r =* 0.65), slopes (*r =* 0.30), and quadratic terms (*r =* 0.55) of weekly alcohol and cannabis use were all significantly correlated, indicating that developmental trajectories in these substances are positively related to one another during adolescence.

**Table 2:** Phenotypic correlations between random intercepts, slopes, and quadratic terms (obtained from latent growth-curve models) with 95% maximum likelihood-based confidence intervals.

Intercept: Intercept correlations				
	Alcohol	Cannabis	Parental monitoring	Home
Alcohol	**1**			
Cannabis	**0.65 (0.58, 0.70)**	**1**		
Parents	**−0.29 (−0.37, −0.19)**	**−0.24 (−0.32, −0.14)**	**1**	
Home	−0.16 (−0.34, 0.02)	−0.06 (−0.26, 0.15)	0.04 (−0.10, 0.18)	**1**
School	−0.11 (−0.29, 0.07)	−0.12 (−0.30, 0.06)	0.00 (−0.14, 0.15)	**0.38 (0.20, 0.53)**
Slope: Slope correlations				
	Alcohol	Cannabis	Parents	Home
Alcohol	**1**			
Cannabis	**0.30 (0.18, 0.42)**	**1**		
Parents	−0.12 (−0.26, 0.02)	−0.14 (−0.29, 0.02)	**1**	
Home	−0.18 (−0.39, 0.03)	**−0.53 (−0.72, −0.25)**	0.21 (−0.04, 0.47)	**1**
School	0.11 (−0.12, 0.32)	0.07 (−0.31, 0.40)	−0.01 (−0.30, 0.29)	0.34 (−0.02, 0.67)
Quadratic: Quadratic correlations				
	Alcohol	Cannabis	Parents	Home
Alcohol	**1**			
Cannabis	**0.55 (0.40, 0.68)**	**1**		
Parents	0.10 (−0.10, 0.30)	−0.06 (−0.33, 0.23)	**1**	
Home	−0.26 (−0.58, 0.08)	**−0.90 (−0.96, −0.74)**	**0.47 (0.08, 0.74)**	**1**
School	0.15 (−0.20, 0.48)	0.44 (−0.36, 0.70)	−0.42 (−0.74, 0.03)	**0.60 (0.23, 0.82)**

Estimated cross-phenotype correlations and 95% confidence intervals between random intercepts, age slopes, and age quadratic effects in latent growth curve models. Included assessments were collected from ages 14 to 18. “Alcohol” and “Cannabis” are log transformed drinks per week and log transformed cannabis uses per week, “Parents” was the maximum parental monitoring score reported by any parental figure at a given timepoint, and “Home” and “School” represent the fraction of time spent at home between midnight and 5 am and the fraction of time spent at school between 8 am and 3 pm. Bolded values indicate correlations where 95% CIs did not include 0.

Our hypothesis that parental monitoring and substance use would be negatively correlated before age 18 was supported at baseline: we found a significant negative correlation of the random intercepts for parental monitoring and both alcohol and cannabis use (*r =* −0.29 to −0.24). However, changes in parental monitoring from ages 14 to 18 were not significantly associated with changes in substance use at this time: no significant correlations were identified between the slopes or quadratic terms for parental monitoring and alcohol or cannabis use (*r* = −0.14 to 0.10, all likelihood-based 95% confidence intervals included 0). Hence, while at age 14, participants who initially experienced higher parental monitoring were likely to experience lower initial levels of substance use, participants who experienced larger changes in parental monitoring during adolescence did not exhibit larger changes in either drinking or cannabis use.

### 3.3. Trajectories of geospatial measures

Turning next to results related to our geospatial measures, after quality control, 7,866,643 unique locations were recorded from 588 twins with a median of 7,956 locations per twin. Location tracking was implemented in the smartphone apps months after recruitment began, which is reflected in the number of locations recorded per twin over time (Supplementary Figure S1B). The rate of location acquisition was otherwise consistent over time, aside from a drop in the second year of remote assessment. One known difference between Android and iOS locations were that the Android location API was designed to record a location approximately every 5 min while the iOS application was designed to record a location only when the twin moved more than 500 m. These patterns are apparent in the distributions of the time and distance between successive points (see Supplementary Figure S2). Consecutive location points were very rarely further apart than 1 day or 100 km. Supplementary Figure S3 shows the distribution of forward filling for iOS locations, consistent with expectations that more forward filling would occur in the middle of the night and on weekdays. Fills that start between 8 PM and 3 AM, between 7 AM and 9 AM, or on Monday through Thursday are longer, reflecting twins’ tendency to move less at night, on weekends, and during the school day.

Time at home and time at school before age 18 showed the expected patterns with time of day and day of week with time at home higher at night than during the day and lower on weekend nights than during the week (Figure 2 and Supplementary Figure S4). Time at school was highest on school days, during school hours and slightly lower on Friday than other school days. Time at school was also much lower on school holidays than other weekdays, supporting the validity of the assessment (Supplementary Figure S5).

The geospatial variables, time at home and time at school, were significantly positively correlated with one another at both the intercept (*r* = 0.38) and quadratic slope levels (*r =* 0.60) while correlations between their linear slopes (*r =* 0.34) were of comparable magnitude but fell just short of statistical significance (results presented in Table 2). These findings indicate developmental trajectories from age 14 to 18 for both going out late at night and being away from school during school hours are positively correlated.

These geospatial variables were hypothesized to represent an alternative measure of the parental monitoring construct during adolescence, but both time at home and time at school were weakly and non-significantly correlated with parental monitoring at both the linear slope and intercept levels (*r* = −0.01 to 0.21, all likelihood-based 95% confidence intervals included 0). There was modest evidence for a relationship at the quadratic level, where parental monitoring was significantly correlated with time at home (*r* = 0.47) and nearly with time at school (*r =* −0.42), though this relationship with time at school was in the opposite of the expected direction and fell short of statistical significance.

While neither time at home nor time at school showed the expected relationships with parental monitoring from age 14–18, time at home (though not time at school) appeared related to substance use. Time at home was not significantly correlated with either alcohol or cannabis use at baseline, though its intercept-level correlation with alcohol use (*r* = −0.16) fell just short of statistical significance. Linear and quadratic changes in time at home showed small to moderate correlations with changes in alcohol use that were not statistically significant (*r =* −0.26 to −0.18) as well as large, statistically significant associations with changes in cannabis use (*r =* −0.53 to −0.90).

### 3.4. Twin-based biometric decomposition analyses

**Figure 3:**
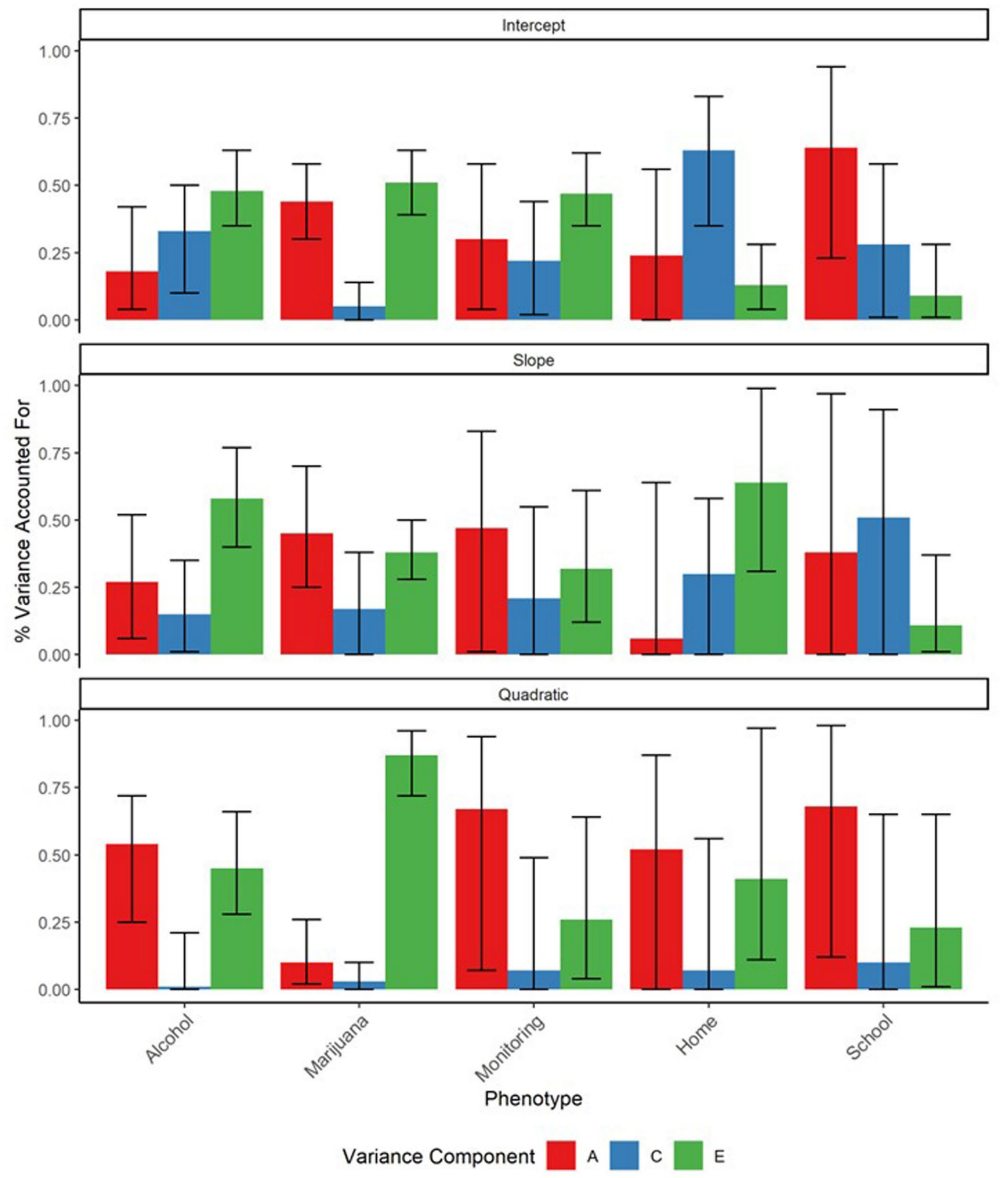
ACE decompositions of the random effects terms for drinks per week, cannabis use per week, parental monitoring, time spent at school, and time spent at home. Substance use phenotypes were aggregated monthly. “A” represents the proportion of variance in the trait attributable to additive genetic effects, “C” represents the proportion attributable to shared environmental effects, and “E” represents the proportion attributable to non-shared environmental effects. Error bars represent 95% maximum likelihood-based confidence intervals.

Biometric ACE decompositions of the random effects, which estimate the contributions of genetic, shared environmental, and nonshared environmental influences to the growth parameters for each phenotype, are reported in Figure 3. Biometric correlations, which tested the extent to which genetic and environmental influences are shared across phenotypes, are presented in Table 3 (unstandardized biometric covariance terms are included as Supplementary Table S1). Due to power constraints, many biometric correlations were estimated with wide confidence bounds, which limited the number of significant effects detected in these models.

**Table 3:** Biometrically decomposed cross-phenotypic correlations between random intercepts, slopes, and quadratic terms (obtained from latent growth-curve models) with 95% maximum likelihood-based confidence intervals.

Intercept: Intercept correlations				
	Alcohol	Cannabis	Parents	Home
Cannabis	***r***_**g**_ **= 0.90**			
	***r***_**c**_ **= 0.85**			
	***r***_**e**_ **= 0.59**			
Parents	*r*_g_ = −0.26	*r*_g_ = −0.35		
	***r***_**c**_ **= −0.61**	*r*_c_ = −0.81		
	*r*_e_ = −0.12	*r*_e_ = −0.03		
Home	*r*_g_ = 0.25	*r*_g_ = −0.46	*r*_g_ = −0.22	
	*r*_c_ = −0.44	*r*_c_ = 0.25	*r*_c_ = 0.09	
	*r*_e_ = −0.07	*r*_e_ = 0.22	*r*_e_ = 0.35	
School	*r*_g_ = 0.40	***r***_**g**_ **= −0.35**	*r*_g_ = −0.48	*r*_g_ = −0.08
	***r***_**c**_ **= −0.79**	*r*_c_ = −0.61	*r*_c_ = 0.55	*r*_c_ = 0.78
	*r*_e_ = −0.02	*r*_e_ = 0.54	***r***_**e**_ **= 0.52**	*r*_e_ = 0.98
Slope: Slope correlations				
	Alcohol	Cannabis	Parents	Home
Cannabis	*r*_g_ = −0.32			
	*r*_c_ = 0.70			
	***r***_**e**_ **= 0.67**			
Parents	*r*_g_ = 0.07	*r*_g_ = 0.01		
	*r*_c_ = 0.18	***r***_**c**_ **= −0.62**		
	***r***_**e**_ **= −0.43**	*r*_e_ = −0.15		
Home	*r*_g_ = −0.55	*r*_g_ = **−1.00**	*r*_g_ = −0.35	
	*r*_c_ = 0.29	*r*_c_ = −0.14	*r*_c_ = 0.42	
	*r*_e_ = −0.29	***r***_**e**_ **= −0.41**	*r*_e_ = 0.47	
School	***r***_**g**_ **= 0.74**	*r*_g_ = 0.52	*r*_g_ = −0.37	*r*_g_ = 0.69
	*r*_c_ = 0.14	*r*_c_ = −0.20	*r*_c_ = 0.35	*r*_c_ = 0.36
	*r*_e_ = −0.69	*r*_e_ = −0.66	*r*_e_ = 0.33	*r*_e_ = 0.15
Quadratic: Quadratic correlations				
	Alcohol	Cannabis	Parents	Home
Cannabis	*r*_g_ = 0.49			
	*r*_c_ = 0.90			
	***r***_**e**_ **= 0.67**			
Parents	*r*_g_ = 0.30	*r*_g_ = −0.17		
	*r*_c_ = −0.29	*r*_c_ = −0.06		
	*r*_e_ = −0.22	*r*_e_ = −0.09		
Home	*r*_g_ = −0.35	*r*_g_ = −0.89	*r*_g_ = 0.27	
	*r*_c_ = 0.15	*r*_c_ = −0.91	*r*_c_ = 0.04	
	*r*_e_ = −0.19	***r***_**e**_ **= −0.92**	***r***_**e**_ **= 0.29**	
School	***r***_**g**_ **= 0.73**	*r*_g_ = 0.77	***r***_**g**_ **= −0.72**	*r*_g_ = 0.66
	*r*_c_ = −0.85	*r*_c_ = 0.74	*r*_c_ = 0.40	*r*_c_ = 0.73
	***r***_**e**_ **= −0.84**	*r*_e_ = 0.81	*r*_e_ = −0.16	*r*_e_ = 0.79

Cross-phenotype biometric correlations obtained from ACE decomposed latent growth curve models. Included assessments were collected from ages 14 to 18. Biometric correlations represent the degree to which genetic, shared environmental, and nonshared environmental influences are shared between phenotypes. “Alcohol” and “Cannabis” are log transformed drinks per week and log transformed cannabis uses per week, “Parents” was the maximum parental monitoring score reported by any parental figure at a given timepoint, and “Home” and “School” represent the fraction of time spent at home between midnight and 5 am and the fraction of time spent at school between 8 am and 3 pm. Bolded values indicate correlations where 95% confidence intervals did not include 0.

Consistent with expectations, baseline alcohol and marijuana use and their developmental trajectories were all significantly heritable (A = 0.10–0.54). Shared environmental factors only significantly contributed to baseline alcohol use (C = 0.33). Similarly, initial levels and developmental changes in parental monitoring were significantly heritable (A = 0.30–0.67), indicating that an adolescent’s reported level of parental monitoring is in part influenced by their genes. Shared environmental influences contributed modestly to baseline parental monitoring (C = 0.22) while non-shared environmental influences made moderate contributions to all three parental monitoring growth parameters (E = 0.26–0.47).

We did not find support for our hypothesis that relationships between parental monitoring and substance use would in part reflect gene–environment correlation. We found no significant genetic correlations for either of the significant, intercept-level relationships between parental monitoring and substance use, or for any of the other (nonsignificant) parental monitoring-substance use relationships. Hence, while we found that both parental monitoring and substance use were significantly influenced by genetic effects, we did not find evidence that genetic correlation significantly contributed to relationships between them. Instead, the significant relationship between baseline alcohol use and parental monitoring was found to be largely explained by the shared environment (*r*_c_ = −0.61), which accounted for 68% of the covariance between alcohol and parental monitoring intercepts. Hence, we found evidence that the baseline relationship between parental monitoring and alcohol use largely reflected mutual shared environmental influences. However, due in part to power constraints, none of the biometric correlations between baseline cannabis use and parental monitoring were significant.

Lastly, biometric analyses also revealed that the significant slope-level relationship between time spent at home and cannabis use was accounted for by mutual genetic (*r*_g_ = −1.00) and nonshared environmental (*r*_e_ = −0.41) influences, with genetic factors accounting for 9% and nonshared environmental factors for 88% of their relationship. At the quadratic level, only this nonshared-environmental component remained significant (*r*_e_ = −0.92), accounting for 93% of the relationship between the cannabis and time at home quadratic terms. These results suggest that adolescents who went out at night more as they grew older also increased their cannabis use and that this relationship in part reflects mutual genetic and non-shared environmental influences on these processes. These participants may also have, to a lesser extent, increased their alcohol use, though these relationships were non-significant in these models. We did not observe similar relationships between substance use and time at school.

## 4. Discussion

In this study, we investigated whether changes in adolescent-reported parental monitoring, a popular intervention target in parent management training interventions, are associated with corresponding changes in adolescent substance use and the extent to which genetic variation contributes to the observed relationship between parental monitoring and substance use. To do so, in a sample of 670 twins during mid-to-late-adolescence, we assessed fine-grained changes in substance use, parental monitoring, and two novel geospatial variables, time spent at home overnight and time spent at school during school hours, which were hypothesized to provide additional information on the parental monitoring construct.

Prior work has shown that substance use can be measured using ecological momentary assessment, such as weekly questions (42, 43), but previous studies of adolescent substance use development have typically had a frequency of assessment measured every few years or used a single occasion of measurement. In contrast, our approach provided much more frequent measurements of substance use over the course of 2 years. More frequent measurements allowed us to evaluate how constructs may change together over time, beyond evaluation of simple difference scores. This advantage is particularly important in studying adolescent substance use behavior due to the rapid changes in substance use behavior seen during this period and its importance to the development of substance use throughout the lifespan (44). Additionally, high frequency GPS-based location data are potentially powerful because it can be linked to other data sets with geographic information, such as maps with place information. These passively collected measures are less susceptible to reporting biases inherent to self-report and thus may be useful in augmenting self-report-based measures of constructs like parental monitoring.

In accord with previous studies, we found that adolescent substance use rates increase dramatically during high school, and additionally found decreases in average levels of parental monitoring, time at school, and time at home over the same period (44, 45). A notable property of all these behaviors is an inflection after age 18 (Figure 2), likely reflecting adolescent maturation and increases in autonomy.

Initial levels of substance use at age 14 (i.e., the random intercept) were correlated with initial level of parental monitoring, though changes in parental monitoring were not significantly related to changes in substance use from ages 14 to 18. The lack of significant correlations between changes in these behaviors fails to support the hypothesized causal effect of parental monitoring on adolescent substance use leveraged by many popular substance use interventions. Nonetheless, significant baseline-associations between these constructs at age 14 suggest there may be such a relationship in early adolescence. We thus cannot rule out that parental monitoring is an important protective factor in early adolescence, and thus may remain a valuable intervention target for delaying substance use, even if later changes in parental monitoring during mid-to-late adolescence are less effective.

Regarding whether genetic confounders influence the relationship between parental monitoring and substance use, we found that substance use and parental monitoring phenotypes were heritable traits. Parental monitoring, though conceived of as an aspect of the adolescent’s environment, was generally found to be even more heritable than substance use was. This is consistent with previous studies on the heritability of parental monitoring, which, at least outside of disadvantaged environments, have found considerable genetic contributions to parental monitoring behaviors in childhood and adolescence (46, 47). This finding may reflect the effect of other heritable behavioral traits, like adolescent personality or parental closeness, on the level of parental monitoring that they experience. The finding that parental monitoring is heritable does not mean the trait is immutable, or particularly resistant to intervention, though the heritability statistic is occasionally misinterpreted in this fashion (48). Indeed, many highly heritable traits are readily susceptible to interventions (eyeglasses for astigmatism, or mood stabilizing medications for bipolar disorder are two such examples); thus, the heritability of parental monitoring has little implication for whether parent management trainings may effectively improve parental monitoring.

Furthermore, though both substance use and parental monitoring were heritable, we did not detect significant genetic correlations between them. Thus, we did not find evidence that genetic confounding underlies the intercept-level relationship we observed between parental monitoring and substance use. Contrastingly, we did find a significant shared (r_c_) environmental correlation underlying this relationship. One interpretation of this is that parental monitoring represents an environmental influence on baseline substance use. Alternatively, aspects of the shared environment, like sociodemographic characteristics, or school and neighborhood effects, may simultaneously influence both substance use and parental monitoring in early adolescence.

**Figure 4:**
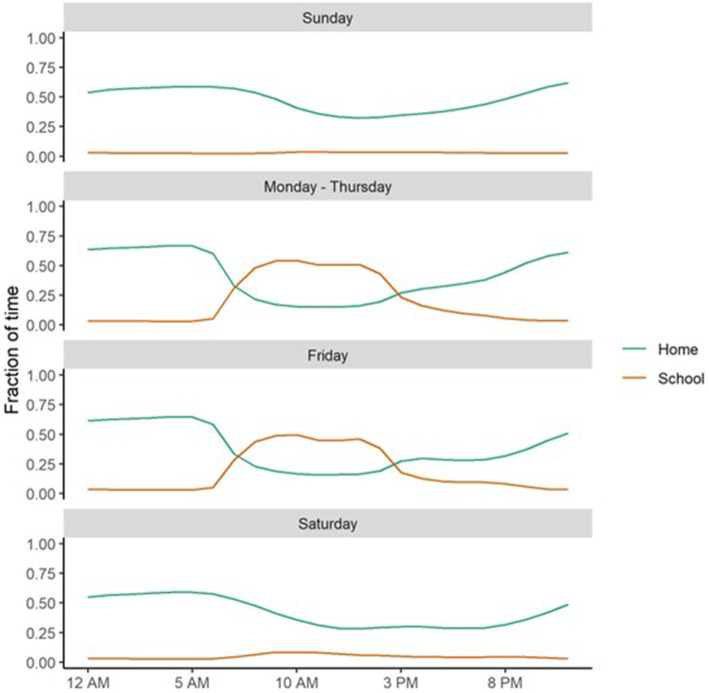
The fraction of time spent at home at night (Home) and at school, during school hours, on school days (School), conditional on time of day and day of week.

The geospatial measures showed the expected relationships with the day of the week, the hour of the day (Figure 4), and the Colorado public school calendar (Supplementary Figure S5), evidencing substantial measurement validity. Contrary to our hypothesis and despite the apparent validity of these geospatial measures, evidence for a relationship between parental monitoring and either time spent at home or at school was weak. This result suggests that adolescents who go out late at night more often or who are more likely to miss school during the day report similar levels of parental monitoring as their peers who engage in lower levels of these behaviors.

Given our hypothesis that these variables measure discordance between a twin’s actual and expected location, this finding is counterintuitive. One explanation is that participants may be disclosing these incidents to their parents, in which case engaging in them more frequently would not impact parental monitoring. Alternatively, it is possible that this time at home variable may also be capturing events unrelated to parental monitoring. These may include overnight stays with friends or relatives or, particularly for the children of divorced parents, at alternative home addresses not provided to the study. Similarly, adolescents may spend less time at school during school hours for many reasons, such as illness or homeschooling that are also unrelated to parental monitoring. Regardless, the lack of associations observed between parental monitoring and these geospatial variables suggest that they are likely not appropriate measures of the parental monitoring construct, at least when it is adolescent reported. To better understand the behavioral constructs that underlie these geospatial measures, additional research on their behavioral correlates is needed.

Time spent at home, though not initially correlated with substance use, showed a strong, negative correlation with changes in cannabis use that was explained by mutual genetic and nonshared environmental influences. This included a perfect −1.00 genetic correlation between the slopes of cannabis and time spent at home, suggesting a strong overlap between the genetic influences on increasing substance use and increased time spent out at night. Thus, the processes influencing whether adolescents go out late at night more as they grow older, which may reflect behaviors like sneaking out late at night to attend parties or see friends, may be strongly influenced by genetic factors associated with risk taking behaviors like substance use.

Due in part to the novel data collection effort, several limitations are noteworthy. First, we required that study participants have a smartphone. While smartphone ownership is true for most youths aged 14–17, it is not universal. This inclusion criterion no doubt contributed to the ethnic and socioeconomic characteristics of the sample. Second, several factors may have contributed to measurement error or bias in the computation of our geospatial variables. While the location API provided estimates of point accuracy, these were not externally verifiable and so include some degree of measurement error. The large size of many suburban and rural high schools in Colorado may have resulted in some miss-classification of GPS points. Additionally, our measure of time at home at night is likely to be downwardly biased in families where a child sometimes stays with relatives or in families where the parents do not live together, as our set of home addresses for a family may not include all homes for those twins. Hence, measures of time at home and time at school may in part reflect behaviors, like attending a large high school or frequently visiting relatives that are less relevant to the parental monitoring construct. This may in part explain their low correspondence with adolescent-reported parental monitoring. Third and relatedly, parental monitoring was adolescent reported rather than parent-reported in this study. While parent and child-reported parental monitoring were positively correlated, they were only moderately so. It is thus possible that adolescent perceptions of parental monitoring may not be fully capturing the true extent of their parents’ knowledge of their activities. Fourth, remote parental monitoring was measured via a subset of the parental monitoring items regarding perceived parental knowledge of their child’s activities. Highly influential research on the parental monitoring construct has previously highlighted that parental monitoring is influenced by two additional constructs: parental solicitation and child disclosure, which were not assessed at the remote follow up assessments (15). Because of this, we are unable to say whether our baseline relationships between substance use and parental monitoring are driven more by parental solicitation efforts or by adolescent self-disclosure to their parents.

Fifth, this study assesses real-world developmental changes in parental monitoring and substance use in a community sample. Though we failed to find corresponding changes in substance use and parental monitoring, the processes driving such changes in our sample may differ in important ways from those involved in parental management training programs, where changes in parental monitoring are induced via intervention and which are carried out in populations with clinically significant substance use or other behavioral difficulties. It is hence possible that their relationship may differ in heavier users or when parental monitoring is undergoing intervention, though additional theory would need to be developed and tested to understand why this would occur. Lastly, this study was conducted in a sample of 670 twins, which was underpowered to detect smaller genetic or environmental correlations, especially when the relevant variance components were small (41). This likely contributed to the wide confidence bounds around ACE estimates and the small number of significant biometric correlations. Hence it is possible that additional genetic and environmental correlations relevant to these relationships were not observed here due to power constraints.

With these limitations in mind, the present study has significant implications for our understanding of the relationship between parental monitoring and substance use. Namely that, at least in community samples, changes in parental monitoring are largely uncorrelated with changes in substance use in mid-to-late adolescence. This suggests that researchers should further explore whether parental monitoring is truly an effective intervention target in substance use interventions in this age group. While meta-analytic work supports the efficacy of parent-management training programs for substance use, additional work may be needed to understand the active ingredients driving these treatments. Further testing the theory underlying these treatments and conducting dismantling studies aimed at isolating their mechanisms of action will help enhance our understanding of these popular interventions and allow for more efficacious, cost-effective treatments in the future.

## Data availability statement

The raw data supporting the conclusions of this article will be made available by the authors, without undue reservation.

## Ethics statement

The studies involving human participants were reviewed and approved by the University of Minnesota and University of Colorado institutional review boards. Written informed consent to participate in this study was provided by the participants’ legal guardian/next of kin.

## Author contributions

JA: conceptualization, data management, methodology, analytic strategy, statistical programming and analyses, wrote manuscript draft, and visualization of results. SF: statistical consulting, data management, and reviewed and edited the manuscript. SZ: statistical consulting, statistical programming, and reviewed and edited the manuscript. RC: grant oversight, initial data collection, project administration, data management, and reviewed and edited the manuscript. AL: data collection, data management, project administration, and reviewed and edited the manuscript. RS: data collection, project administration, and reviewed and edited the manuscript. CP and HS: data management and reviewed and edited the manuscript. MF and SR: data collection, data management, and reviewed and edited the manuscript. GR-S, MS, and HV: data collection and reviewed and edited the manuscript. MJ and QZ: grant oversight and reviewed and edited the manuscript. YL, ML, MM, SW, and PR: reviewed and edited the manuscript. JH: grant oversight, data management, and reviewed and edited the manuscript. NF: grant oversight, project conceptualization, supervision, statistical consulting, and reviewed and edited the manuscript. SV: grant oversight, project conceptualization, primary supervision, statistical consulting, and reviewed and edited the manuscript. All authors contributed to the article and approved the submitted version.

## Funding

This work was funded separately by T32DA050560 to SV and NF. This grant funds the Colorado Twin Study (COTwins), a multi-year, smartphone-based intensive longitudinal assessment study on the genetic and environmental contributors to the development of adolescent substance use and related behaviors and the research and training of GR-S, a contributing author to this manuscript (T32DA050560 to GR-S).

## Conflict of interest

The authors declare that the research was conducted in the absence of any commercial or financial relationships that could be construed as a potential conflict of interest.

## Publisher’s note

All claims expressed in this article are solely those of the authors and do not necessarily represent those of their affiliated organizations, or those of the publisher, the editors and the reviewers. Any product that may be evaluated in this article, or claim that may be made by its manufacturer, is not guaranteed or endorsed by the publisher.

## Footnotes

^1^ https://nces.ed.gov/ccd/elsi/tableGenerator.aspx
