# Supplementary material for: Evaluating longitudinal relationships between parental monitoring and substance use in a multi-year, intensive longitudinal study of 670 adolescent twins
Source: Front Psychiatry. 2023 May 12;14:1149079. doi: 10.3389/fpsyt.2023.1149079 (PMC10213319; doi:10.3389/fpsyt.2023.1149079)
Supplement: Supplementary file 1 [file Data_Sheet_1.docx]

**Supplementary Tables:**

| **Table S1. Raw ACE decomposed Variances and Covariances from Latent Growth Models** | | | | | | |
| --- | --- | --- | --- | --- | --- | --- |
|  | |  |  |  |  |  |
| Random Intercepts | | | | | | |
|  | Alcohol | | Cannabis | Parents | Home | School |
| Alcohol | **V = 0.069**  **A = 0.002**  **C = 0.029**  **E = 0.038** | |  |  |  |  |
| Cannabis | **Cov_V_ = 0.037**  **Cov_A_ = 0.021**  Cov_C_ = 0.000  **Cov_E_ = 0.016** | | **V = 0.112**  **A = 0.068**  C = 0.000  **E = 0.044** |  |  |  |
| Parents | **Cov_V_ = -0.138**  Cov_A_ = 0.005  **Cov_C_ = -0.094**  Cov_E_ = -0.049 | | **Cov_V_ = -0.191**  Cov_A_ = -0.114  Cov_C_ = -0.071  Cov_E_ = -0.006 | **V = 4.796**  **A = 1.329**  **C = 1.244**  **E = 2.223** |  |  |
| Home | Cov_V_ = -0.013  Cov_A_ = 0.001  Cov_C_ = -0.003  Cov_E_ = -0.011 | | Cov_V_ = -0.065  Cov_A_ = -0.038  Cov_C_ = 0.001  Cov_E_ = -0.027 | Cov_V_ = 0.033  Cov_A_ = -0.002  Cov_C_ = 0.004  Cov_E_ = 0.031 | **V = 0.029**  A = 0.001  **C = 0.022**  **E = 0.006** |  |
| School | Cov_V_ = 0.001  Cov_A_ = 0.005  **Cov_C_ = -0.006**  Cov_E_ = 0.003 | | Cov_V_ = -0.038  **Cov_A_ =** -**0.035**  Cov_C_ = 0.004  Cov_E_ = -0.007 | Cov_V_ = -0.016  Cov_A_ = 0.044  Cov_C_ = -0.019  **Cov_E_ = -0.041** | **Cov_V_ = 0.016**  Cov_A_ = 0.011  Cov_C_ = 0.001  Cov_E_ = 0.004 | **V = 0.025**  **A = 0.016**  C = 0.008  E = 0.001 |
|  |  | |  |  |  |  |
| Random Age Effects | | | | | | |
|  | Alcohol | | Cannabis | Parents | Home | School |
| Alcohol | **V = 9.040**  **A = 5.027**  C = 0.011  **E = 4.003** | |  |  |  |  |
| Cannabis | **Cov_V_ = 16.637**  Cov_A_ = 3.333  Cov_C_ = 0.086  **Cov_E_ = 12.449** | | **V = 60.970**  **A = 6.869**  C = 2.227  **E = 51.875** |  |  |  |
| Parents | Cov_V_ = 6.426  Cov_A_ = 11.584  Cov_C_ = -0.750  **Cov_E_ = -4.408** | | Cov_V_ = -10.535  Cov_A_ = -7.424  **Cov_C_ = 4.010**  Cov_E_ = -7.121 | **V = 424.311**  **A = 280.494**  C = 30.102  **E = 113.714** |  |  |
| Home | Cov_V_ = -2.014  Cov_A_ = -1.543  Cov_C_ = -0.143  Cov_E_ = -0.328 | | **Cov_V_ = -56.280**  **Cov_A_ = -5.287**  Cov_C_ = -1.311  **Cov_E_ = -49.685** | Cov_V_ = 46.747  Cov_A_ = 15.943  Cov_C_ = 5.552  Cov_E_ = 25.251 | **V = 16.049**  A = 6.961  C = 2.437  **E = 6.652** |  |
| School | Cov_V_ = 1.642  **Cov_A_ = 5.120**  Cov_C_ = -0.357  Cov_E_ = -3.120 | | Cov_V_ = 19.577  Cov_A_ = 7.902  Cov_C_ = 0.587  Cov_E_ = 11.088 | Cov_V_ = -33.204  Cov_A_ = -34.157  Cov_C_ = 4.290  Cov_E_ = -3.338 | Cov_V_ = 8.941  Cov_A_ = 6.296  Cov_C_ = 0.704  Cov_E_ = 1.942 | **V = 14.764**  A = 9.973  C = 1.216  E = 3.575 |
|  |  | |  |  |  |  |
| Random Age^2^ Effects | | | | | | |
|  | Alcohol | | Cannabis | Parents | Home | School |
| Alcohol | **V = 10.099**  **A = 0.573**  C = 0.005  **E = 4.521** | |  |  |  |  |
| Cannabis | **Cov_V_ = 15.837**  Cov_A_ = 3.174  Cov_C_ = 0.695  **Cov_E_ = 11.969** | | **V = 58.584**  **A = 5.643**  C = 1.971  **E = 50.971** |  |  |  |
| Parents | Cov_V_ = 6.810  Cov_A_ = 12.332  Cov_C_ = -0.345  Cov_E_ = -5.177 | | Cov_V_ = -9.434  Cov_A_ = -5.794  Cov_C_ = 3.467  Cov_E_ = -7.107 | **V = 472.351**  **A = 315.558**  C = 34.307  **E = 122.485** |  |  |
| Home | Cov_V_ = -2.427  Cov_A_ = -1.732  Cov_C_ = -0.044  Cov_E_ = -0.651 | | **Cov_V_ = -53.439**  Cov_A_ = -4.612  Cov_C_ = -1.113  **Cov_E_ = -49.686** | **Cov_V_ = 48.498**  Cov_A_ = 15.426  Cov_C_ = 4.979  **Cov_E_ = 28.093** | **V = 16.964**  A = 6.894  C = 2.762  **E = 7.308** |  |
| School | Cov_V_ = 1.926  **Cov_A_ = 5.576**  Cov_C_ = -0.345  **Cov_E_ = -3.305** | | Cov_V_ = 18.426  Cov_A_ = 7.098  Cov_C_ = 0.466  Cov_E_ = 10.862 | Cov_V_ = -35.145  **Cov_A_ = -36.572**  Cov_C_ = 4.573  Cov_E_ = -3.146 | **Cov_V_ = 9.224**  Cov_A_ = 6.559  Cov_C_ = 0.673  Cov_E_ = 1.992 | **V = 15.661**  **A = 10.602**  C = 0.503  **E = 3.555** |
| Growth Parameter Means | | | | | | |
|  | Alcohol | | Cannabis | Parents | Home | School |
| Intercept | 0.138 | | 0.078 | 16.351 | 0.061 | -0.058 |
| Age | -0.551 | | -0.216 | 4.139 | -1.541 | 1.706 |
| Age^2^ | 0.650 | | 0.289 | -5.060 | 1.503 | -1.852 |

Unstandardized variances, cross-phenotypic covariances, and genetic, shared environmental, and non-shared environmental variance components acquired from multivariate growth models. “Alcohol” and “Cannabis” are log transformed drinks per week and log transformed cannabis uses per week, “Parents” was the maximum parental monitoring score reported by any parental figure at a given timepoint, and “Home” and “School” represent the fraction of time spent at home between midnight and 5am and the fraction of time spent at school between 8am and 3pm. Notably, this does not represent a single simultaneously estimated covariance matrix. Rather, values for covariances were derived from five separate bivariate models and presented together here. Bolded values indicate that 95% maximum-likelihood based confidence intervals did not include zero.

**Supplementary Figures:**

**Figure S1.** The per-person rate of data acquisition by time since enrollment in the study, binned by week, for A) the remote surveys, with a green line showing the maximum number of surveys possible in any given week if each twin completed every survey immediately after the survey was scheduled. Please note that most surveys would not even be noticed until the twin received a reminder notification, which would only be pushed the following day. The peak at year 1.0 may reflect a participant desire to receive their 1-year honorarium, thereby catching up on surveys. In plot B) Android and iOS location frequencies are shown. The Android and iOS software necessary to collect locations was under development at the start of the study and was completed for Android before iOS, leading to the observed lag in B and the continual increase seen for iOS up until year 2 was complete.


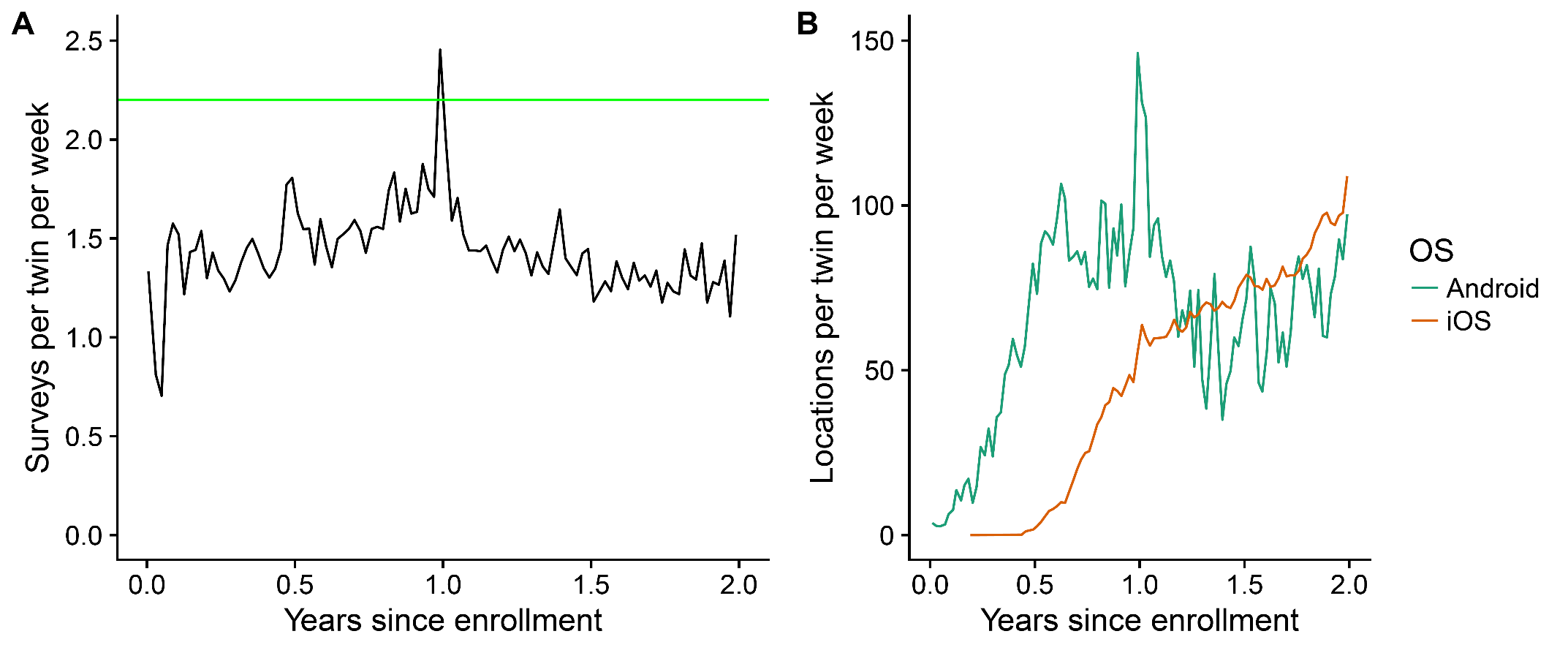


**Figure S2**. Stacked histograms of the intervals in A) time and B) space between consecutive locations for a twin, conditional on the type of phone used by the twin. The expected patterns are seen. First, the Android time intervals center around five minutes and have less variance than the iOS time intervals. Second, most iOS space intervals are 500 meters or larger and Android space intervals are much more likely to be small or zero. The larger distances (e.g., 1 kilometer or larger), especially for iOS, were observed during piloting when the participant was driving in a car. Particularly large distances (e.g., >100 kilometers) were observed, in some cases, between airports, indicating the individual had just traveled by airplane.
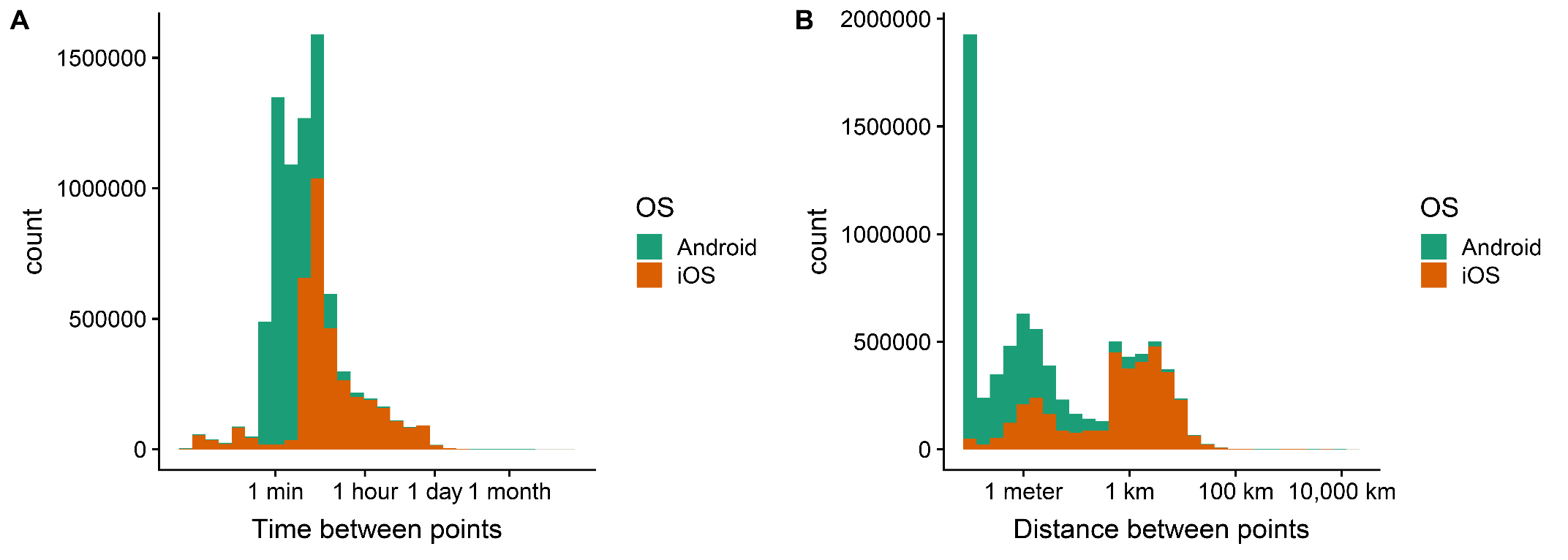


**Figure S3**. Boxplots of the length in hours of the forward fills of the iOS location data, conditional on A) the hour of the day, and B) the day of the week, of the point being filled forward. Outliers are not displayed to avoid overplotting. As expected, fills starting at night or at the start of the school day are longer, indicating that twins are less likely to move late at night and during the school day. Fills starting Monday through Thursday are longer than those starting on Friday, Saturday, or Sunday, indicating that twins move more overnight on Friday, Saturday, and Sunday. This is consistent with twins spending less time at home and at school on weekends.


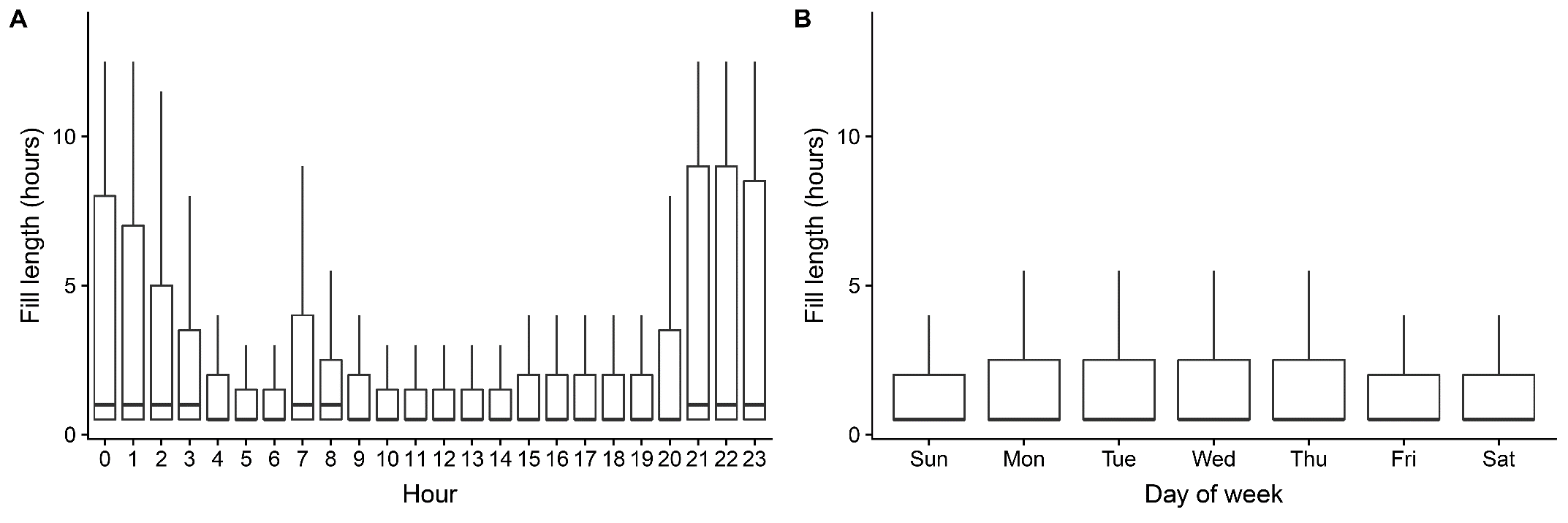


**Figure S4**. Heatmaps showing the fraction of filled and standardized points recorded before age 18 that were A) within 100 meters of a home address for a given twin, or B) that were within 200 meters of a Colorado high school on a school day, conditional on the day of the week and the hour of the day. As expected, participants are more likely to be at home at night than during the day and on weekday nights than on weekend nights. Twins are less likely to be at home during school hours on school days than on weekends. Participants tend to be at school during school hours on weekdays. They also spend less time at school on Friday than the other weekdays.


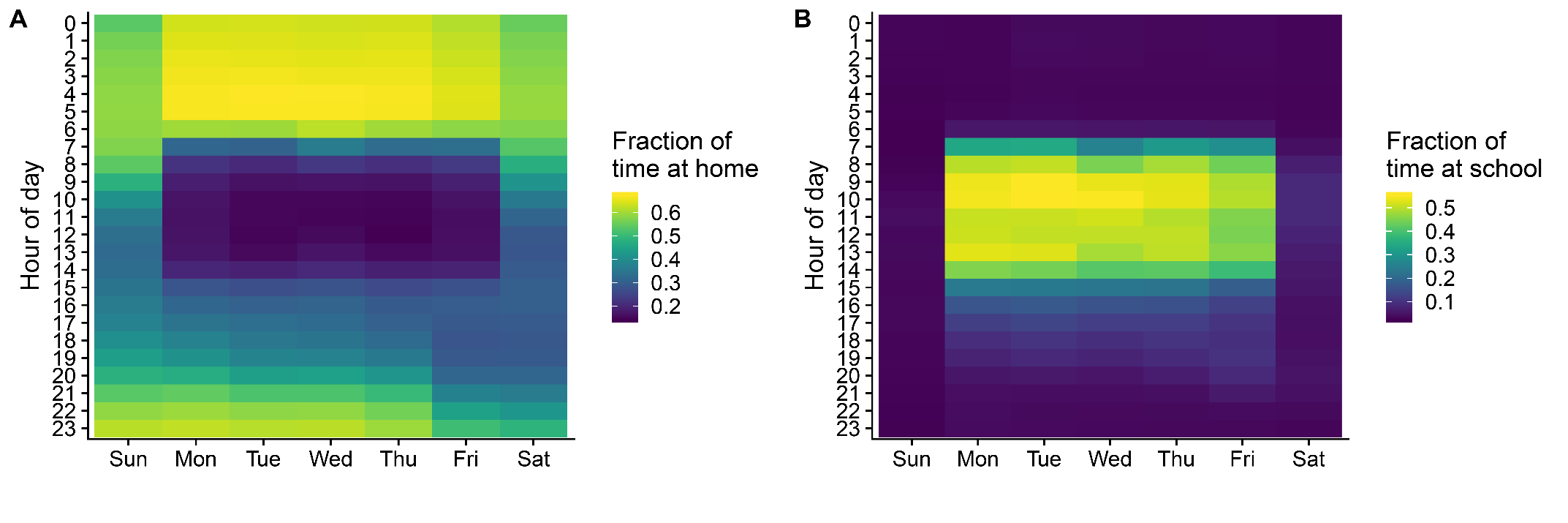


**Figure S5**. A calendar heatmap showing the fraction of filled and standardized points recorded before age 18 that were within 200 meters of a Colorado high school, for each calendar day. Weekends, summer months, and school holidays consistently show a much lower fraction of points at school.
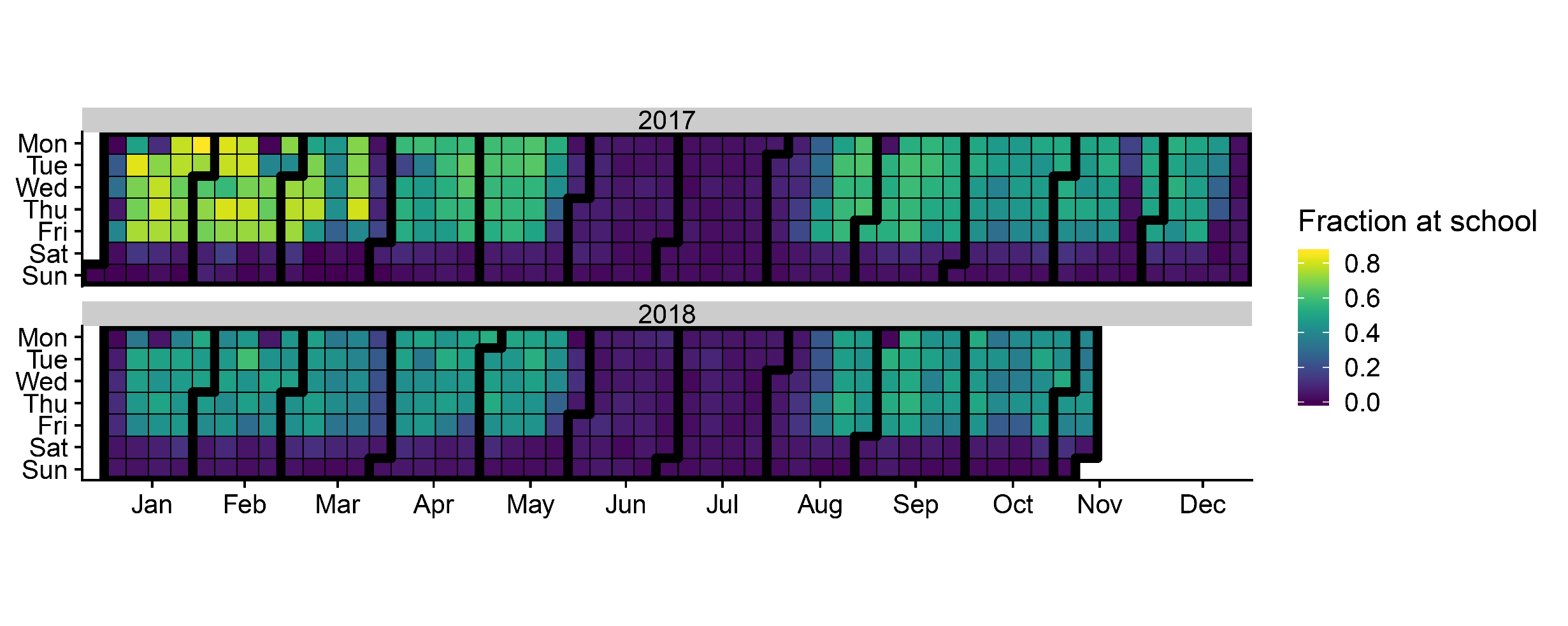


**Appendix A: Parental monitoring questionnaire**
