# Supplementary material for: Evaluating longitudinal relationships between parental monitoring and substance use in a multi-year, intensive longitudinal study of 670 adolescent twins
Source: Front Psychiatry. 2023 May 12;14:1149079. doi: 10.3389/fpsyt.2023.1149079 (PMC10213319; doi:10.3389/fpsyt.2023.1149079)
Supplement: Supplementary file 2 [file Data_Sheet_2.pdf]

We'd like to ask you how much your parents know about different aspects of your life. We will ask how often your parents actually know about these aspects of your life. So think about what you tell them, as well as what they find out from other sources, such as talking with other parents, monitoring phone calls, and so on.

A1. Who are the adults you live with?

- Father ☐
- Stepfather ☐
- Mother ☐
- Stepmother ☐
- Other Adult ☐
- I don't live with any of these adults ☐
- Would rather not answer ☐

A2. We'd like to ask you how much these individuals know about different aspects of your life. We will ask how often each person you checked actually knows about these aspects of your life. So think about what you tell them, as well as what they find out from other sources, such as talking with other parents, monitoring phone calls, and so on.

- Got it! ☐

B1. My mother knows who I spend time with.

- Never ☐
- Once in a while ☐
- Sometimes ☐
- Often ☐
- Always ☐
- Don't know ☐
- Prefer not to answer ☐

B2. **My mother knows how I spend my money.**

- Never ☐
- Once in a while ☐
- Sometimes ☐
- Often ☐
- Always ☐
- Don't know ☐
- Prefer not to answer ☐

B3. **My mother knows where I am most afternoons after school.**

- Never ☐
- Once in a while ☐
- Sometimes ☐
- Often ☐
- Always ☐
- Don't know ☐
- Prefer not to answer ☐

B4. **My mother knows where I go at night.**

- Never ☐
- Once in a while ☐
- Sometimes ☐
- Often ☐
- Always ☐
- Don't know ☐
- Prefer not to answer ☐

B5. **My mother knows what I do with my free time.**

- Never ☐
- Once in a while ☐
- Sometimes ☐
- Often ☐
- Always ☐
- Don't know ☐
- Prefer not to answer ☐

C1. **My father knows who I spend time with.**

- Never ☐
- Once in a while ☐
- Sometimes ☐
- Often ☐
- Always ☐
- Don't know ☐
- Prefer not to answer ☐

C2. **My father knows how I spend my money.**

- Never ☐
- Once in a while ☐
- Sometimes ☐
- Often ☐
- Always ☐
- Don't know ☐
- Prefer not to answer ☐

C3. **My father knows where I am most afternoons after school.**

- Never ☐
- Once in a while ☐
- Sometimes ☐
- Often ☐
- Always ☐
- Don't know ☐
- Prefer not to answer ☐

C4. **My father knows where I go at night.**

- Never ☐
- Once in a while ☐
- Sometimes ☐
- Often ☐
- Always ☐
- Don't know ☐
- Prefer not to answer ☐

C5. **My father knows what I do with my free time.**

- Never ☐
- Once in a while ☐
- Sometimes ☐
- Often ☐
- Always ☐
- Don't know ☐
- Prefer not to answer ☐

D1. **My stepfather knows who I spend time with.**

- Never
- Once in a while
- Sometimes
- Often
- Always
- Don't know
- Prefer not to answer

D2. **My stepfather knows how I spend my money.**

- Never
- Once in a while
- Sometimes
- Often
- Always
- Don't know
- Prefer not to answer

D3. **My stepfather knows where I am most afternoons after school.**

- Never
- Once in a while
- Sometimes
- Often
- Always
- Don't know
- Prefer not to answer

D4. **My stepfather knows where I go at night.**

- Never ☐
- Once in a while ☐
- Sometimes ☐
- Often ☐
- Always ☐
- Don't know ☐
- Prefer not to answer ☐

D5. **My stepfather knows what I do with my free time.**

- Never ☐
- Once in a while ☐
- Sometimes ☐
- Often ☐
- Always ☐
- Don't know ☐
- Prefer not to answer ☐

E1. **My stepmother knows who I spend time with.**

- Never ☐
- Once in a while ☐
- Sometimes ☐
- Often ☐
- Always ☐
- Don't know ☐
- Prefer not to answer ☐

E2. **My stepmother knows how I spend my money.**

- [ ] Never
- [ ] Once in a while
- [ ] Sometimes
- [ ] Often
- [ ] Always
- [ ] Don't know
- [ ] Prefer not to answer

E3. **My stepmother knows where I am most afternoons after school.**

- [ ] Never
- [ ] Once in a while
- [ ] Sometimes
- [ ] Often
- [ ] Always
- [ ] Don't know
- [ ] Prefer not to answer

E4. **My stepmother knows where I go at night.**

- [ ] Never
- [ ] Once in a while
- [ ] Sometimes
- [ ] Often
- [ ] Always
- [ ] Don't know
- [ ] Prefer not to answer

E5. **My stepmother knows what I do with my free time.**

- Never ☐
- Once in a while ☐
- Sometimes ☐
- Often ☐
- Always ☐
- Don't know ☐
- Prefer not to answer ☐

F1. **In the first question when we asked which adults you lived with, you checked "other adult". Please briefly describe who this other adult is.**

F2. **The other adult I live with knows who I spend time with.**

- Never ☐
- Once in a while ☐
- Sometimes ☐
- Often ☐
- Always ☐
- Don't know ☐
- Prefer not to answer ☐

F3. **The other adult I live with knows how I spend my money.**

- Never ☐
- Once in a while ☐
- Sometimes ☐
- Often ☐
- Always ☐
- Don't know ☐
- Prefer not to answer ☐

F4. **The other adult I live with knows where I am most afternoons after school.**

- [ ] Never
- [ ] Once in a while
- [ ] Sometimes
- [ ] Often
- [ ] Always
- [ ] Don't know
- [ ] Prefer not to answer

F5. **The other adult I live with knows where I go at night.**

- [ ] Never
- [ ] Once in a while
- [ ] Sometimes
- [ ] Often
- [ ] Always
- [ ] Don't know
- [ ] Prefer not to answer

F6. **The other adult I live with knows what I do with my free time.**

- [ ] Never
- [ ] Once in a while
- [ ] Sometimes
- [ ] Often
- [ ] Always
- [ ] Don't know
- [ ] Prefer not to answer
