# Supplementary material for: Evaluating longitudinal relationships between parental monitoring and substance use in a multi-year, intensive longitudinal study of 670 adolescent twins
Source: Front Psychiatry. 2023 May 12;14:1149079. doi: 10.3389/fpsyt.2023.1149079 (PMC10213319; doi:10.3389/fpsyt.2023.1149079)
Supplement: Supplementary file 3 [file Data_Sheet_3.pdf]

A1. In the last 7 days, since last {date('l')}, on how many days did you drink any alcohol?

- I did not drink any alcohol in the past 7 days ☐
- One day ☐
- Two days ☐
- Three days ☐
- Four days ☐
- Five days ☐
- Six days ☐
- Seven days ☐
- Would rather not answer ☐

A2. On those days that you drank alcohol, how many drinks did you usually have each day? (One "drink" is equal to 1 can or bottle of beer, a glass of wine, or a shot of hard liquor.)

- Only part of one drink ☐
- 1 drink ☐
- 2 drinks ☐
- 3 drinks ☐
- 4 drinks ☐
- 5 drinks ☐
- 6 drinks (such as a 6-pack of beer or 6 shots of liquor) ☐
- 7 drinks ☐
- 8 drinks ☐
- 9 drinks ☐
- 10 drinks ☐
- 11 drinks ☐
- 12 drinks (such as a 12-pack of beer or 12 glasses of wine) ☐
- 13 drinks ☐
- 14 drinks ☐
- 15 drinks ☐
- 16 drinks ☐
- 17 drinks ☐
- 18 drinks ☐
- 19 drinks ☐
- 20 drinks ☐
- More than 20 drinks ☐
- Would rather not answer ☐

B1. **In the last 7 days, since last {date('l')}, on how many days did you use any marijuana or hashish, including smoking marijuana, edibles, vaping, dabbing, or however else you may have used marijuana?**

- I did not have any marijuana in the past 7 days ☐
- One day ☐
- Two days ☐
- Three days ☐
- Four days ☐
- Five days ☐
- Six days ☐
- Seven days ☐
- Would rather not answer ☐

B2. **On each day that you used marijuana (whether smoked, eaten, vaped, dabbed, or however it was used), how many times per day did you use enough to feel the effects?**

- I never had enough to feel the effects ☐
- 1 time per day ☐
- 2 times per day ☐
- 3 times per day ☐
- 4 times per day ☐
- 5 times or more per day ☐
- Would rather not answer ☐
